# Supplementary material for: A high-quality draft genome assembly of the Neotropical butterfly, Batesia hypochlora (Nymphalidae: Biblidinae)
Source: BMC Genomics. 2025 Dec 6;27:31. doi: 10.1186/s12864-025-12394-z (PMC12797539; doi:10.1186/s12864-025-12394-z)
Supplement: Supplementary file 1 — Supplementary Material 1. [file 12864_2025_12394_MOESM1_ESM.pdf]

Supplementary data

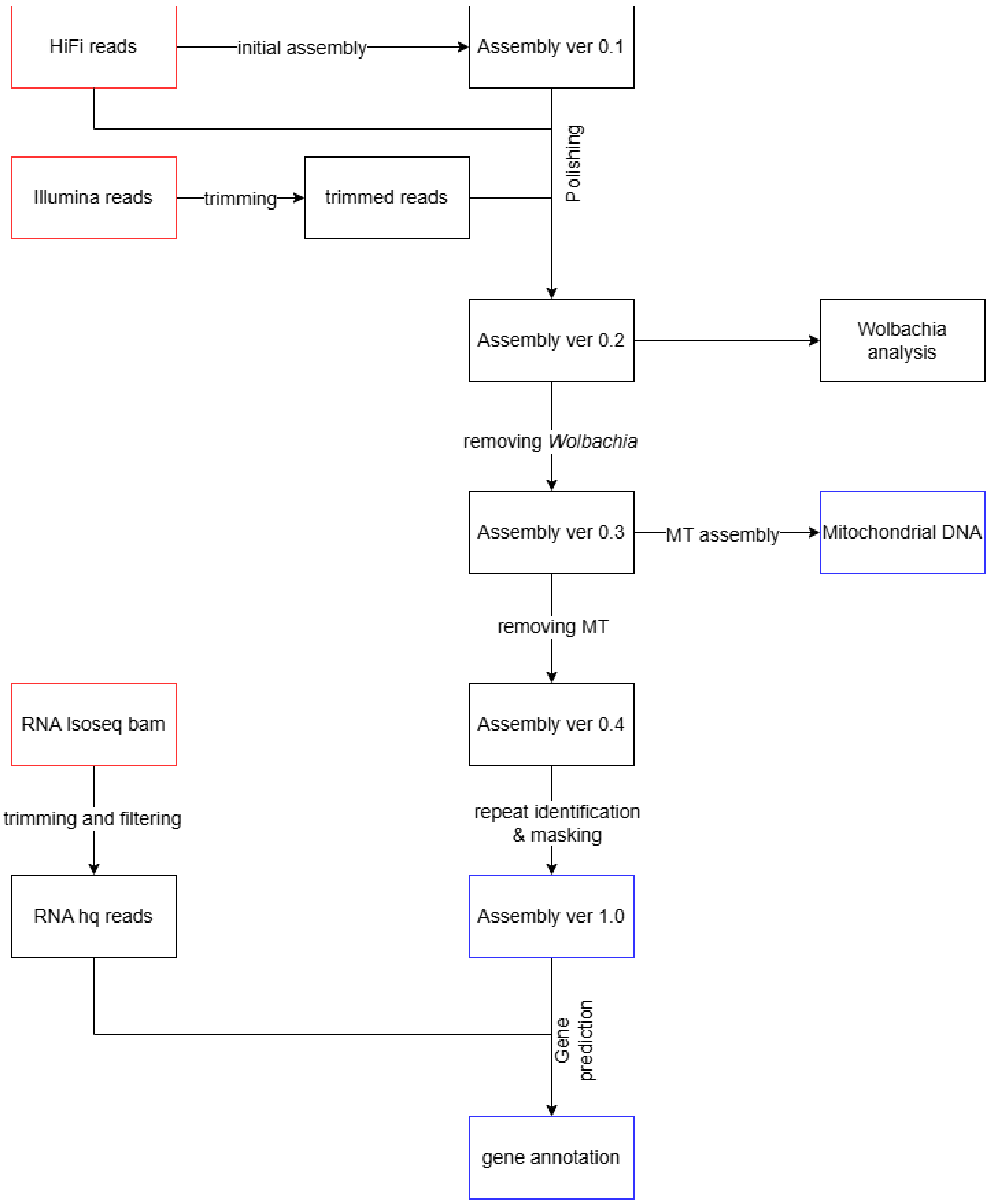

Figure S1. **Workflow of *B. hypochlora* assembly**. The red boxes are the sequence inputs, and the blue boxes are the outputs, which are available on the NCBI database

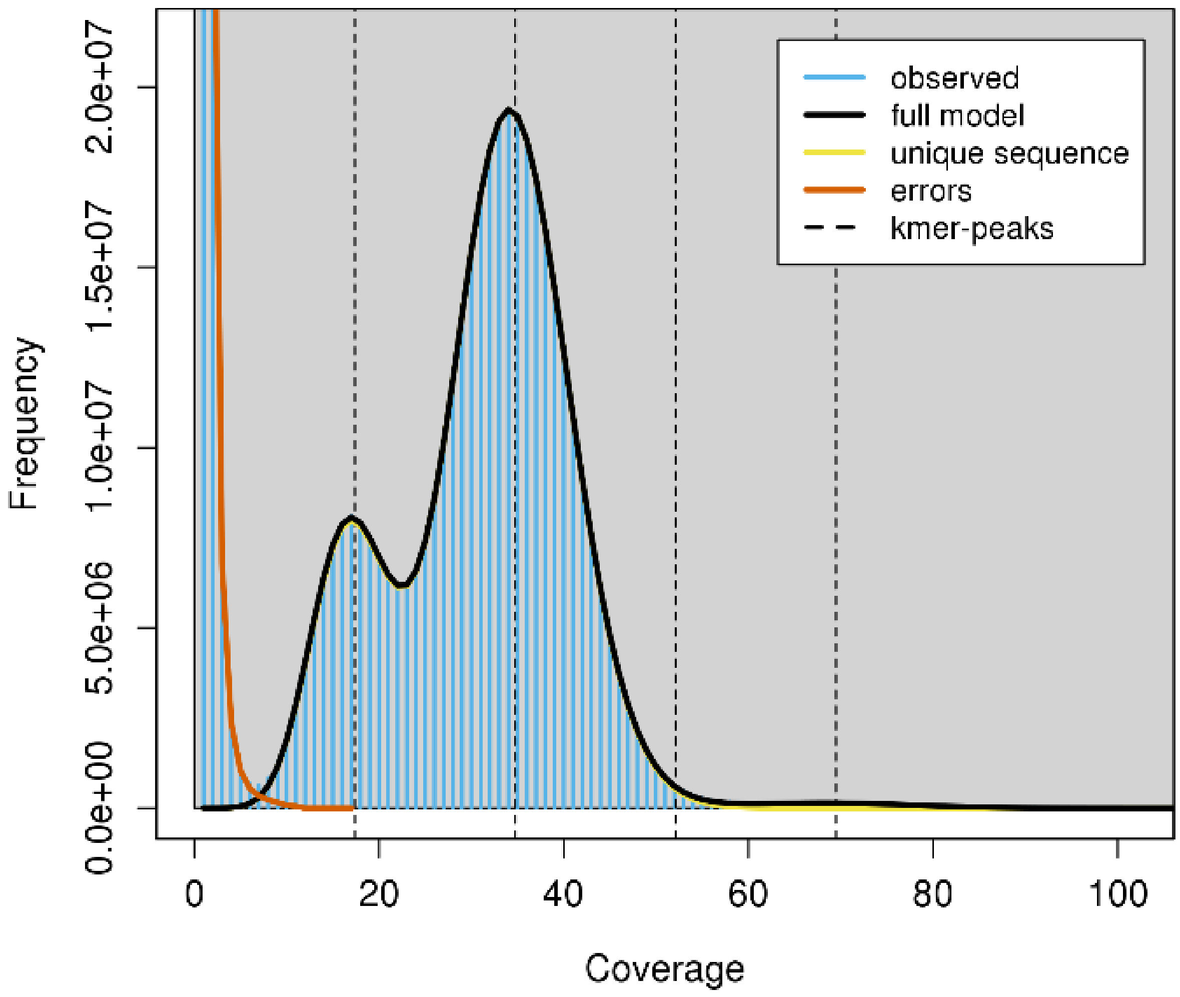

Figure S2. **Kmer analysis of unassembled PacBio HiFi reads**. Kmer frequency distribution (k=31) indicates an estimated genome size of 379.39 Mbp, with a diploid coverage of 17.4 X and a low heterozygosity (0.429%).

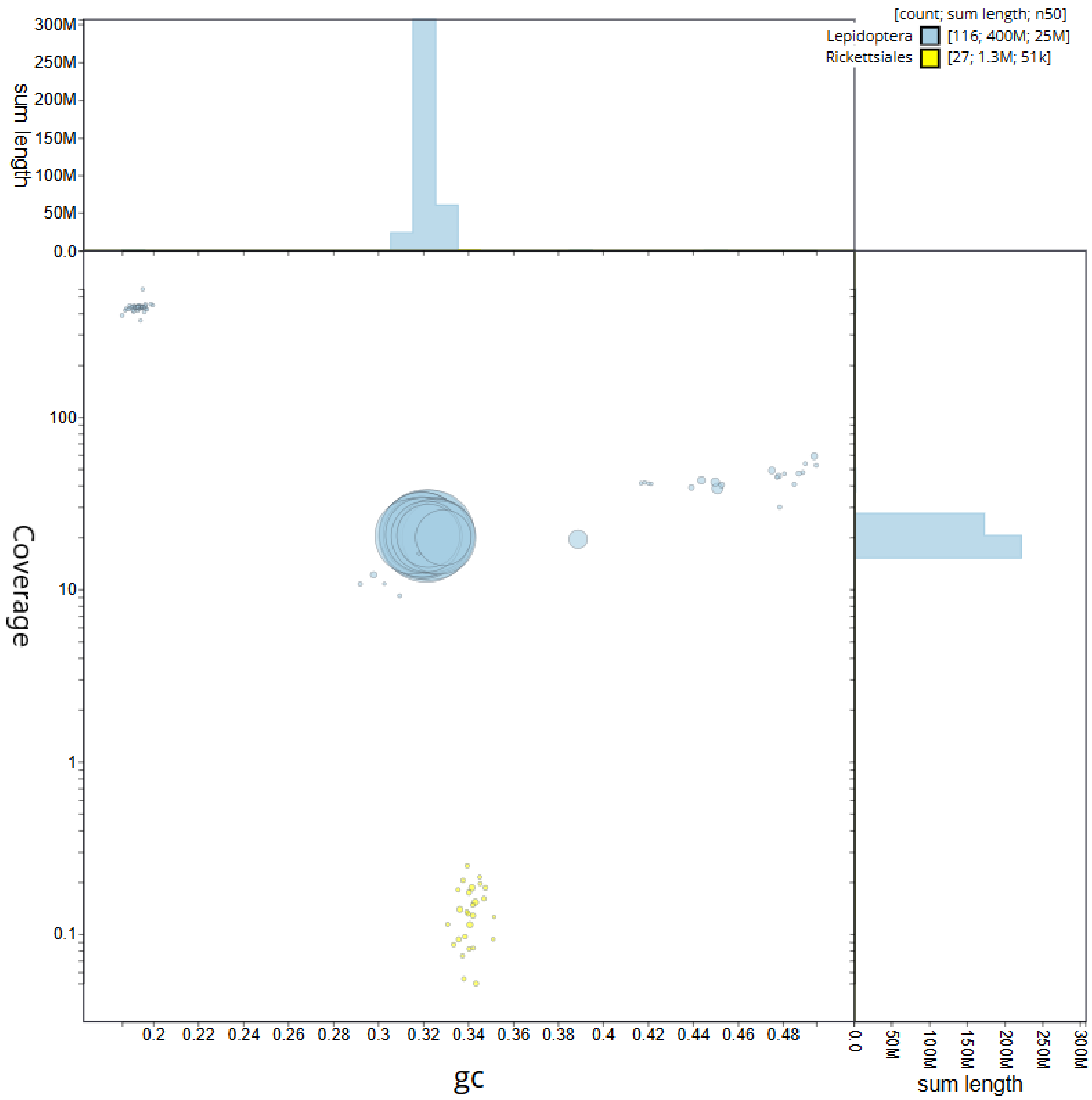

Figure S3. **Blob plot of base coverage in alignment against GC content for contigs in genome version 0.2.** Sequences are colored by taxonomic annotation at the family level. Circles are sized in proportion to sequence length on a square-root scale, ranging from 12,666 bp to 37,552,201 bp, and yellow circles are *Wolbachia* contigs. Histograms show the distribution of the sequence length sum along each axis

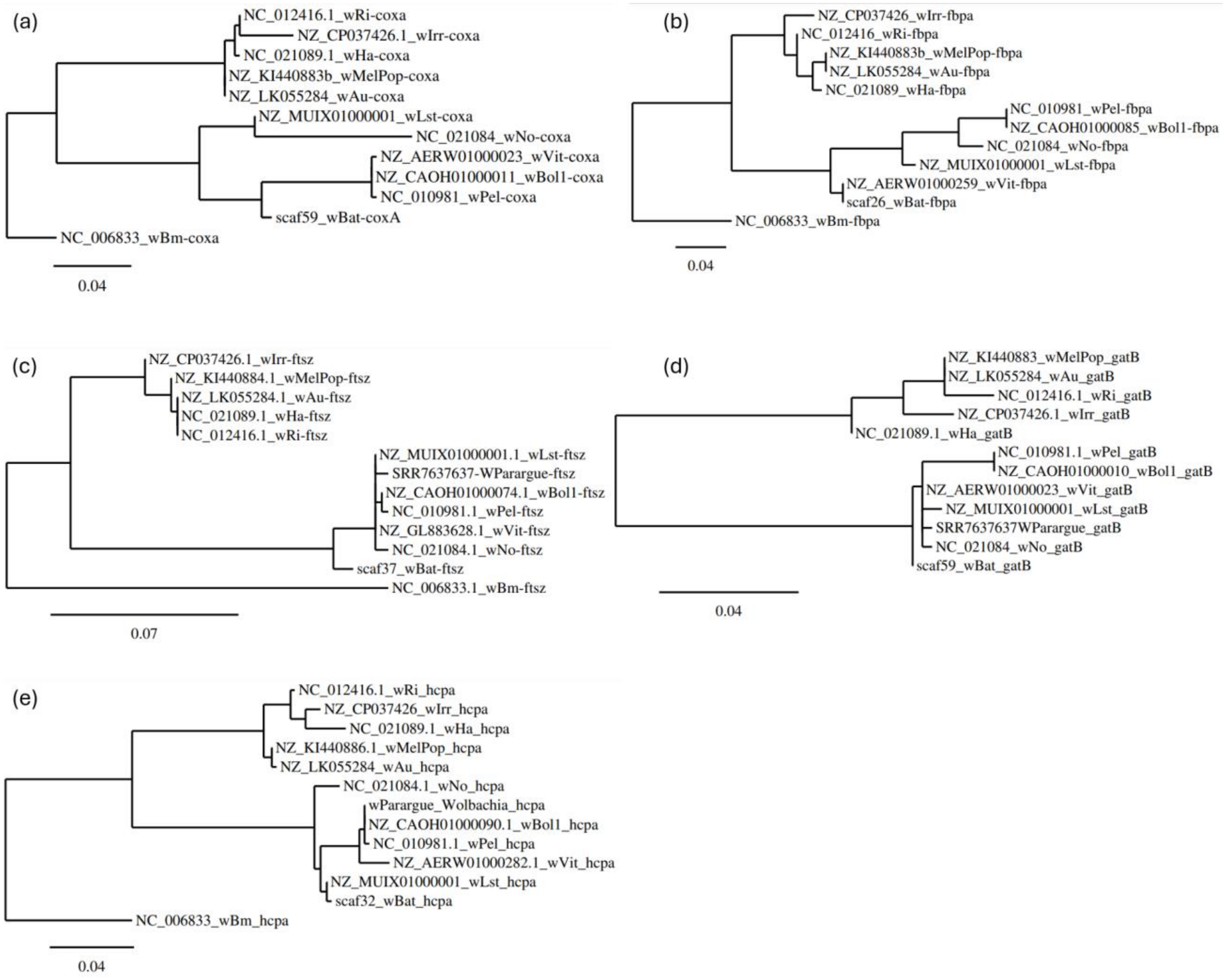

Figure S4. **Phylogeny of *Wolbachia* genes active in Batesia:** (a) *coxa* genes, (b) *fbpa* genes, (c) *ftsz* genes, and (d) *hcpa* genes. The *wmk* gene is not active

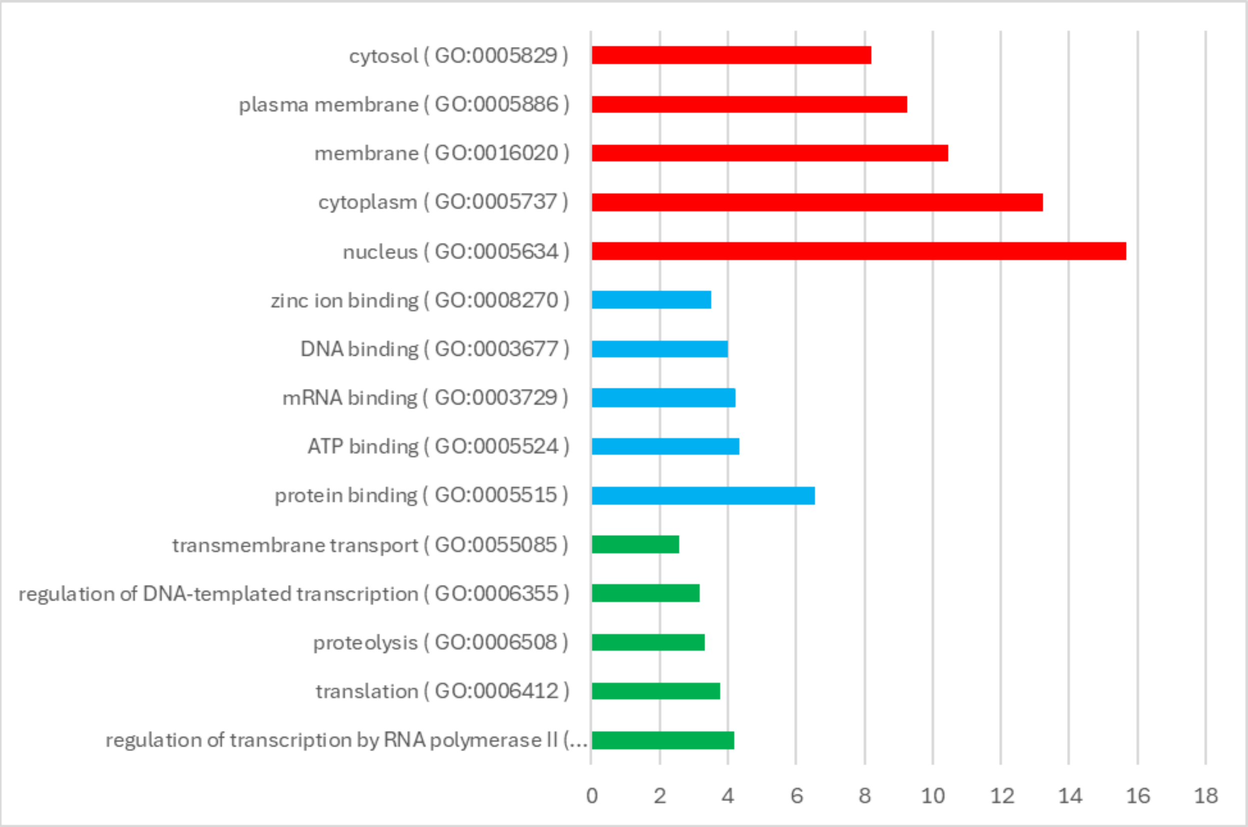

Figure S5. **The five highest frequencies of GO terms are in three ontology aspects**. The red bars represent the cellular component, the blue bars represent molecular function, and the green bars represent the biological process. The x-axis is the percentage of genome frequency of each GO term.

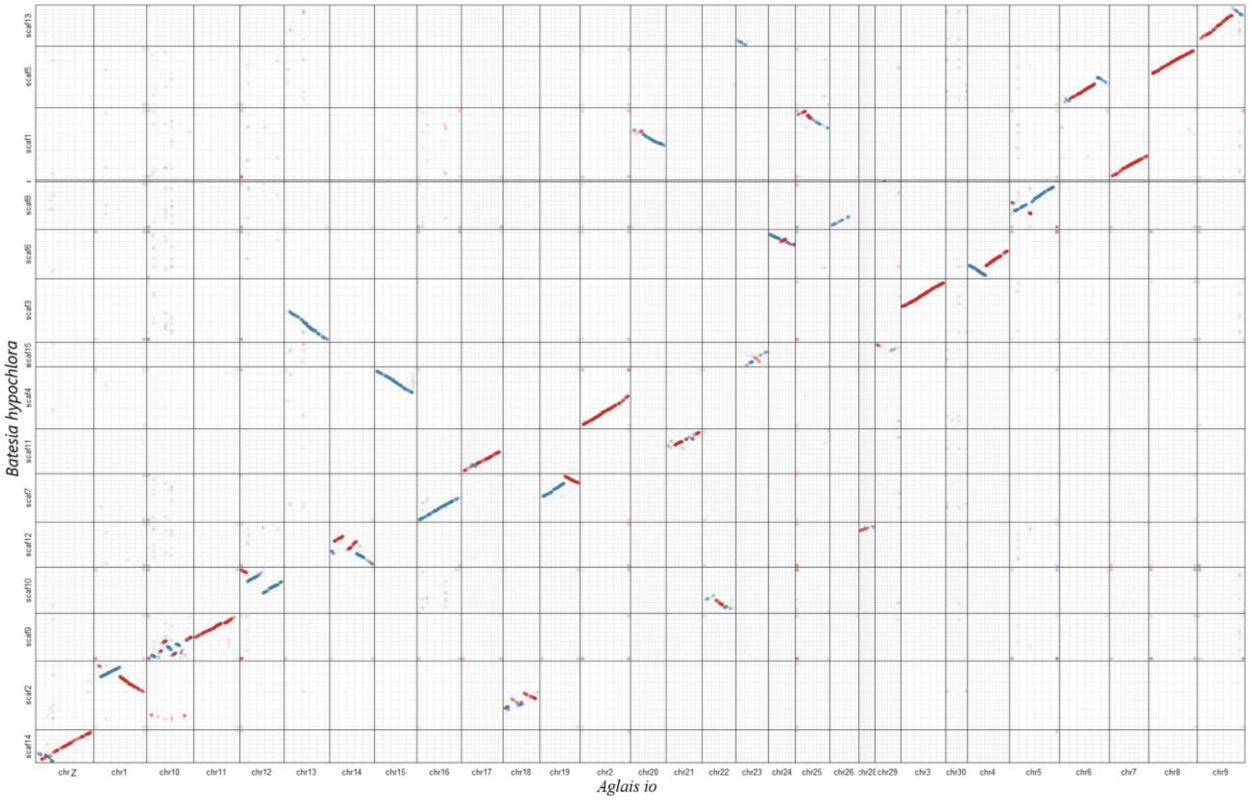

Figure S6. **Chromosome rearrangements between *Batesia hypochlora* and *Aglais io* on the dot plot** show chromosomal fusions between the two species, *B. hypochlora* genome (15 scaffolds on the y-axis) and *A. io* (30 + Z chromosomes on the x-axis). Chromosome 27 of the *A. io* genome is not presented due to a minimal alignment with the *B. hypochlora* genome. Red dots are "+" strand, and blue dots are "-" strand

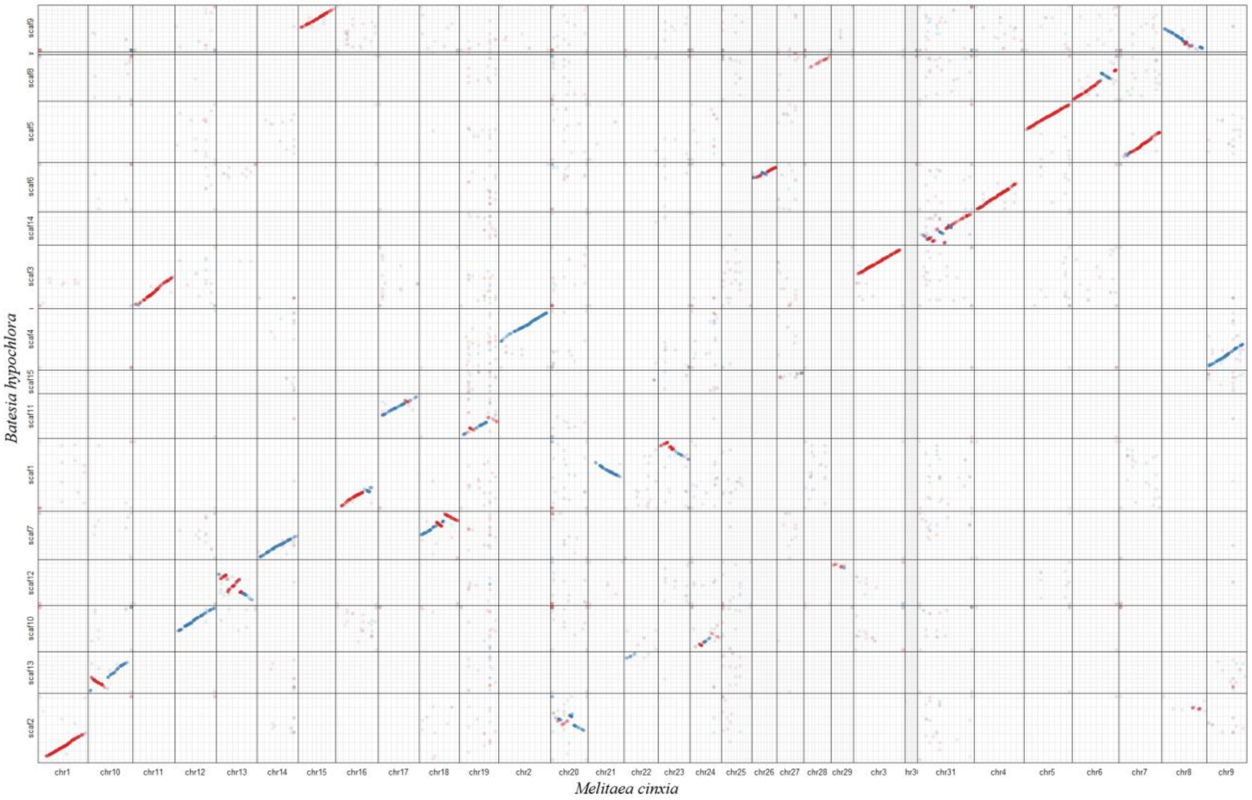

Figure S7. **Chromosome rearrangements between *Batesia hypochlora* and *Melitaea cinxia* on the dot plot** show chromosomal fusions between the two species, *B. hypochlora* genome (15 scaffolds on the y-axis) and *M. cinxia* (30 + Z chromosomes on the x-axis). Two single alignments on scaffold 21 (2,782bp) and scaffold 44 (4,022bp) were not presented. Red dots are "+" strand, and blue dots are "-" strand
